# Modeling effect of the septic condition and trauma on C-reactive protein levels in children with sepsis: a retrospective study

**DOI:** 10.1186/cc5955

**Published:** 2007-06-28

**Authors:** Michal Kyr, Michal Fedora, Lubomir Elbl, Nishan Kugan, Jaroslav Michalek

**Affiliations:** 1 1st Department of Pediatrics, University Hospital Brno, Cernopolni 9, Brno, 61300, Czech Republic; 2 Masaryk University Institute of Biostatistics and Analyses, Brno, Czech Republic; 3 Department of Pediatric Anesthesiology and Resuscitation, University Hospital Brno, Brno, Czech Republic; 4 Department of Cardiopulmonary Testing, University Hospital Brno, Brno, Czech Republic; 5 University of Massachusetts, Worcester, 01655, MA, USA

## Abstract

**Introduction:**

Sepsis is the main cause of morbidity and mortality in intensive care units and its early diagnosis is not straightforward. Many studies have evaluated the usefulness of various markers of infection, including C-reactive protein (CRP), which is the most accessible and widely used. CRP is of weak diagnostic value because of its low specificity; a better understanding of patterns of CRP levels associated with a particular form of infection may improve its usefulness as a sepsis marker. In the present article, we apply multilevel modeling techniques and mixed linear models to CRP-related data to assess the time course of CRP blood levels in association with clinical outcome in children with different septic conditions.

**Methods:**

We performed a retrospective analysis of 99 patients with systemic inflammatory response syndrome, sepsis, or septic shock who were admitted to the Pediatric Critical Care Unit at the University Hospital, Brno. CRP blood levels were monitored for 10 days following the onset of the septic condition. The effect of different septic conditions and of the surgical or nonsurgical diagnosis on CRP blood levels was statistically analyzed using mixed linear models with a multilevel modeling approach.

**Results:**

A significant effect of septic condition and diagnosis on the course of CRP levels was identified. In patients who did not progress to septic shock, CRP blood levels decreased rapidly after reaching peak values – in contrast to the values in patients with septic shock in whom CRP protein levels decreased slowly. Moreover, CRP levels in patients with a surgical diagnosis were higher than in patients with a nonsurgical condition. The magnitude of this additional elevation in surgical patients did not depend on the septic condition.

**Conclusion:**

Understanding the pattern of change in levels of CRP associated with a particular condition may improve its diagnostic and prognostic value in children with sepsis.

## Introduction

Sepsis remains the main cause of morbidity and mortality in intensive care units [1,2]. Host immunodeficiency, increasing bacterial resistance to antibiotics, and problematic discrimination of an early onset of infection are the major factors altering the course of infections [3,4]. Early diagnosis of sepsis and consequently its correct treatment are fundamental to achieving a positive outcome for patients. Many studies have evaluated the usefulness of various markers of infection in different septic conditions – C-reactive protein (CRP), procalcitonin (PCT), TNFα, and IL-6, IL-8, and IL-10 [5-9].

In clinical practice, CRP is the most accessible and widely used marker of infection, and many authors have addressed its sensitivity and specificity [5,10-14], some of whom compared CRP levels among various diagnoses and/or severities of organ dysfunction [13,14]. Various noninfectious insults, such as trauma [15] or malignancy, can influence the levels of inflammatory markers, especially CRP [16] – leading to a decrease in the diagnostic value of CRP. Therefore CRP seems to be a sensitive but less specific marker of infection. Several studies have focused on how CRP levels change over time to improve its diagnostic value [12-14,17,18]; however, hardly any have involved a true longitudinal analysis of the data to assess how various factors affect CRP levels. In our study, we incorporated these considerations and analyzed our data using a multilevel linear model with mixed effects [19-22]. Knowing the factors influencing CRP levels in sepsis as well as the patterns of these levels associated with different medical or surgical conditions can lead to a better understanding of its diagnostic value.

## Materials and methods

### Study population

We performed a retrospective study collecting data from patients 0–18 years old participating in a gene polymorphism study [23]. All pediatric patients whose parents or legal guardians gave informed consent approved by an Institutional Ethics Committee were included. Inclusion criteria for participation in the study included admission to the pediatric critical care unit at the University Hospital Brno, Brno, Czech Republic, for at least 24 hours and a presence of systemic inflammatory response syndrome (SIRS), sepsis, severe sepsis, septic shock, or multiple organ dysfunction syndrome (MODS), defined according to the consensus conference [24]. Patients admitted to the pediatric critical care unit from September 2003 to December 2005 were enrolled.

If a patient was admitted to the pediatric critical care unit more than once, only the first admission was considered. Each patient was assessed for a septic condition each day of the hospital stay. CRP blood levels were recorded, if present, using a turbidimetry technique with a Hitachi 917 (Roche Diagnostics, Basel, Switzerland) device. Each patient was classified according to the presence of infection and to the most severe septic condition that developed over the 10-day period: noninfectious group (NIN), comprising SIRS, shock, or MODS of noninfectious origin; septic group (SPT), comprising sepsis or severe sepsis; or septic shock or MODS group (SSM) in the presence of infection. The international pediatric sepsis consensus criteria were used for patient classification [24]. The 10-day period was considered as follows: for NIN patients, day 0 was the first day of SIRS being present; in patients with infection (SPT and SSM patients), day 0 was considered the first day of SIRS in the course of infection. Patients were further classified as surgical (major surgery or trauma immediately preceding the septic condition) or nonsurgical.

### Statistical analysis

We used a graphical analysis to explore the dynamics of CRP levels and to help identify the final model used. A logarithmic transformation of the response variable 'CRP level' was performed to achieve an approximately normal distribution, and these transformed data were used in the analyses. A longitudinal data analysis was performed using mixed models and multilevel modeling techniques. Unconditional means and growth models, as well as two final conditional models, are presented here. For the terminology of unconditional models we refer to Singer and Willett [19]. Table 1 provides the model specifications.

**Table 1:** Model specifications

Model	Level 1	Level 2
Unconditional means model	*Y*_*ij *_= β_0*i *_+ *e*_*ij*_	β_0*i *_= γ_00 _+ *u*_0*i*_
Unconditional growth model	*Y*_*ij *_= β_0*i*_+ β_1*i*_TIME_*ij *_+ β_2*i *_TIME_ij_*TIME_*ij *_+ *e*_*ij*_	β_0*i *_= γ_00 _+ *u*_0*i*_
		β_1*i *_= γ_10 _+ *u*_1*i*_
		β_2*i *_= γ_20 _+ *u*_2*i*_
Model A	*Y*_*ij *_= β_0*i*_+ β_1*i*_TIME_*ij *_+ β_2*i *_TIME_ij_*TIME_*ij *_+ *e*_*ij*_	β_0*i *_= γ_00 _+ γ_01_SEP_*i *_+ *u*_0*i*_
		β_1*i *_= γ_10 _+ γ_11_SEP_*i *_+ *u*_1*i*_
		β_2*i *_= γ_20 _+ *u*_2*i*_
Model B	*Y*_*ij *_= β_0*i *_+ β_1*i*_TIME_*ij *_+ β_2*i *_TIME_ij_*TIME_*ij *_+ *e*_*ij*_	β_0*i *_= γ_00 _+ γ_01_SEP_*i *_+ γ_02_DG_*i *_+ *u*_0*i*_
		β_1*i *_= γ_10 _+ γ_11_SEP_*i *_+ *u*_1*i*_
		β_2*i *_= γ_20 _+ *u*_2*i*_

**Figure 1:**
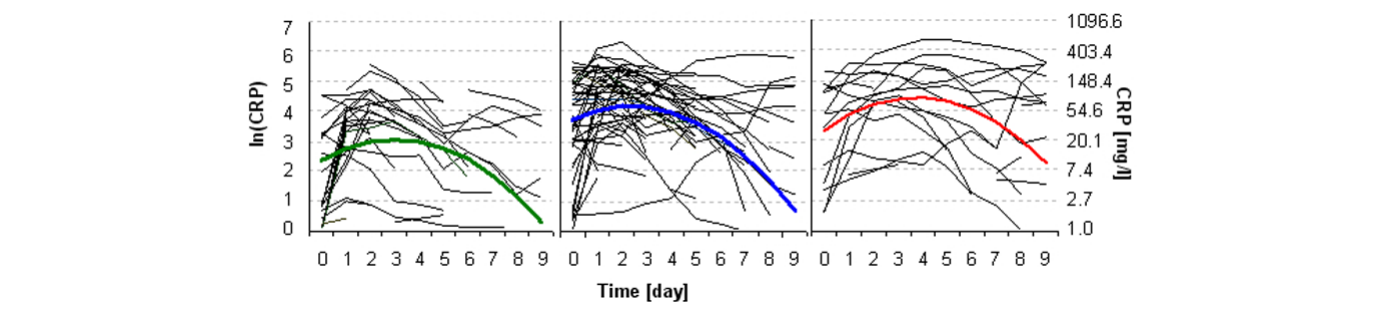
Individual C-reactive protein curves. Individual level (thin lines) and model level (bold lines) C-reactive protein (CRP) curves of the noninfectious group (left, green), the septic group (middle, blue), and the shock and multiple organ dysfunction syndrome group (right, red).

A two-level mixed linear model was applied. At level 1 of the model, the response variable *Y *= ln(CRP) was considered a quadratic function of time with random parameters for each patient. We selected the quadratic function based on the exploratory data analysis presented in Figure 1. At level 2 of the model, the random parameters from model level 1 were explained using a variance analysis model with fixed effects. Two parameter models at level 2, *A *and *B*, were considered according to the number of factors involved. Only one factor, category of septic condition (SEP), with three levels (NIN, SPT, SSM), was involved in model A. Two factors, SEP and diagnosis (DG), with two categories (surgical, nonsurgical), were involved in model B.

Table 1 presents the formal notation. Index *i *was used to identify a patient and index *j *to identify a repeated observation in time. The variance components correspond to the variance of the error term *u *from Table 1. The normal distributions of all error terms have been assumed.

Analyses were performed using SAS 9.1 package (SAS Institute Inc., Cary, NC, USA). For mixed modeling, Proc Mixed (SAS Institute Inc.) was used and a maximum likelihood estimation method was adopted. The model fit was evaluated according to Akaike's information criteria and Schwarz's information criteria (smaller values indicate better fit).

## Results

We collected data for a total of 99 patients with sufficient records totaling 588 waves of CRP levels. The mean patient age was 7.6 years (range, 0.1 to 18.5 years). Our sample population consisted of 65 males and 34 females, with 41 surgical patients and 58 nonsurgical patients. The NIN comprised 32 patients who developed SIRS, only two of whom experienced shock. All patients in the SPT had, by definition [24], severe sepsis. In the SSM, 10 patients with septic shock and seven patients with MODS were included. Table 2 summarizes the numbers of patients in each diagnostic group. For more detailed insight into the data, the mean age (standard deviation) and clinical diagnoses of nonsurgical patients according to the group analyzed are summarized in Tables 3 and 4, respectively. The NIN patients were associated with 100% survival of the pediatric critical care unit stay; the SPT patients were associated with 4.2% mortality; and, as expected, the highest mortality (35.3%) occurred in the SSM patients.

**Table 2:** Numbers of patients

	Diagnosis	
	
Category of severity	Internal	Surgical
Noninfectious group	7	27
Sepsis/severe sepsis group	37	11
Septic shock/multiple organ dysfunction syndrome group	14	3
Total	58	41

**Table 3:** Mean (standard deviation) age of patients

	Diagnosis	
	
Category of severity	Internal	Surgical
Noninfectious group	5.6 ± 6.5	8.2 ± 5.8
Sepsis/severe sepsis group	7.2 ± 5.9	10.8 ± 5.8
Septic shock/multiple organ dysfunction syndrome group	5.6 ± 5.8	9.3 ± 0.8

**Table 4:** Diagnoses of nonsurgical patients

Diagnosis	Noninfectious group	Sepsis/severe sepsis group	Septic shock/multiple organ dysfunction syndrome group
Status epilepticus	2	6	2
Central nervous system	0	7	0
Respiratory	0	14	5
Cancer	0	1	4
Other	5	9	3
Total	7	37	14

The fitted models and parameter estimates are presented in Table 5. We present two unconditional and two final models. Because the SEP and DG predictors take on three and two discrete values, respectively, equations for the two full models for respective septic and diagnosis categories can be rewritten as presented in Table 6.

**Table 5:** Models fitted

	Parameter	Unconditional models			Model A	Model B
				
		Means	Growth			
				
			Linear	Quadratic		
**Fixed effects**						
Initial status	Intercept (γ_00_)	3.488***	3.631***	3.215***	2.327***	1.830***
Main effect	SEP (γ_01_)				***	***
	SSM				1.029	1.402
	SPT				1.415	1.762
	NIN				0	0
	DG (γ_02_)					*
	Surgical					0.661
	Nonsurgical					0
Rate of change	TIME (γ_10_)		-0.039^NS^	0.438***	0.483***	0.471***
	TIME*TIME (γ_20_)			-0.077***	-0.079***	-0.078***
Factorial	SEP*TIME (γ_11_)				*	*
	SSM				0.101	0.109
	SPT				-0.113	-0.104
	NIN				0	0
	DG*TIME					NS/EX
	SEP*DG					NS/EX
	SEP*DG*TIME					NS/EX
**Random effects (variance components)**						
Level 1	Residuals (var(*e*_*ij*_) = σ^2^)	1.001***	0.044***	0.265***	0.263***	0.263***
Level 2	Intercept (var(*u*_0*i*_) = σ_00_)	1.257***	2.069***	2.579***	2.045***	2.086***
	TIME (var(*u*_1*i*_) = σ_11_)		0.078***	0.541***	0.534***	0.532***
	TIME*TIME (var(*u*_2*i*_)=σ_22_)			0.008***	0.008***	0.008***
Akaike's information criteria		1,875.8	1,769.8	1,566.4	1,544.0	1,539.6
Schwarz's information criteria		1,883.5	1,785.4	1,592.4	1,580.4	1,578.6

NIN, noninfectious group; SPT, septic group; SSM, shock and multiple organ dysfunction syndrome group; SEP = effect of septic category; DG = diagnosis effect. *P < 0.05; ***P < 0.001; NS, not significant; NS/EX, not significant and excluded.

**Table 6:** Equations for the two full models for respective septic and diagnosis categories

Model A	
NIN category	ln(CRP) = 2.327 + 0.483*TIME - 0.079*TIME*TIME
SPT category	ln(CRP) = 2.327 + 1.415 + 0.483*TIME - 0.113*TIME - 0.079*TIME*TIME
SSM category	ln(CRP) = 2.327 + 1.029 + 0.483*TIME + 0.101*TIME - 0.079*TIME*TIME
Model B	
NIN nonsurgical category	ln(CRP) = 1.83 + 0.471*TIME - 0.078*TIME*TIME
NIN surgical category	ln(CRP) = 1.83 + 0.661 + 0.471*TIME - 0.078*TIME*TIME
SPT nonsurgical category	ln(CRP) = 1.83 + 1.762 + 0.471*TIME - 0.104*TIME - 0.078*TIME*TIME
SPT surgical category	ln(CRP) = 1.83 + 1.762 + 0.661 + 0.471*TIME - 0.104*TIME - 0.078*TIME*TIME
SSM nonsurgical category	ln(CRP) = 1.83 + 1.402 + 0.471*TIME + 0.109*TIME - 0.078*TIME*TIME
SSM surgical category	ln(CRP) = 1.83 + 1.402 + 0.661 + 0.471*TIME + 0.109*TIME - 0.078*TIME*TIME

CRP = C-reactive protein; NIN = noninfectious group; SPT = septic group; SSM = shock and multiple organ dysfunction syndrome group.

**Figure 2:**
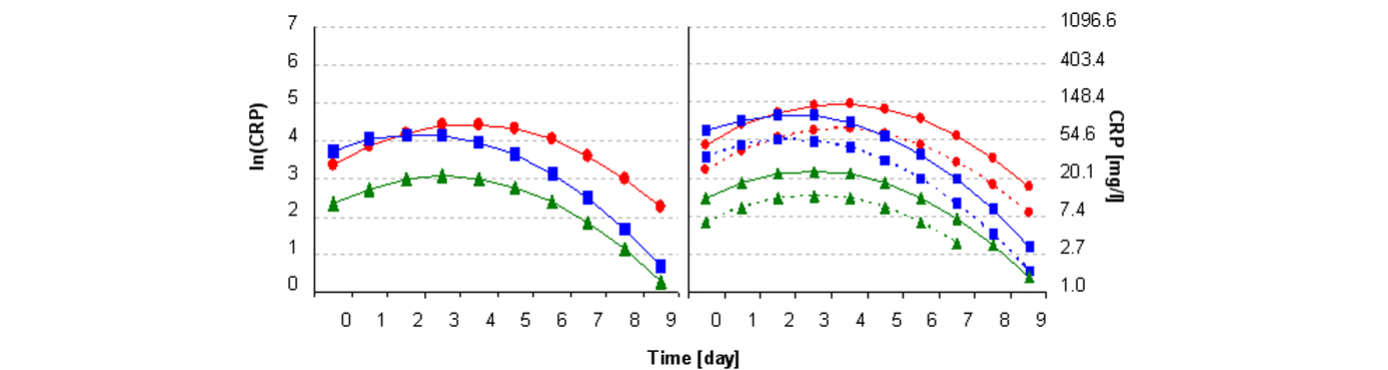
Model curves. Predicted C-reactive protein (CRP) level curves of (left) model A and (right) model B. ▲, Noninfectious group; ■, septic group; and ●, shock and multiple organ dysfunction syndrome group. Model B: nonsurgical patients (dashed lines) and surgical patients (solid lines) conditions.

The graphical representations of the models fitted are shown in comparison with individual data (Figure 1) and in comparison with each septic or diagnosis category (Figure 2). For ease of interpretation, raw values are also presented in the graphs.

## Discussion

### Unconditional models

Fitting unconditional models enables quantification of the overall variance present in our data [19,20]. Including independent variables (predictors) in the model, we can assess the reduction of the variance caused by an included predictor; that is, explained variability accounted for the effect of predictors. First, we fitted an unconditional means and growth model (Table 5). The linear model was not significant as soon as we estimated the intercept. We then identified the quadratic model with significant effects. Comparing residual variance from these two models, we found that a great deal of explainable variation, almost 74%, could be explained by a quadratic level 1 model of time (unconditional growth model). CRP dynamics therefore provide a great deal of information. We then included all level 2 independent variables and their interactions. Estimates of interaction of diagnosis by septic category, diagnosis by time, and diagnosis by septic category by time were not significant predictors (data not shown). Excluding these, we arrive at the two final models (Table 5).

### Full model A

The first model (full model A) includes the septic category (SEP) and the septic category by time interaction (SEP*TIME) as predictors. This model indicates that baseline CRP levels are lowest in the NIN; an average child without infection has a baseline ln(CRP) = 2.33, peaking at approximately day 3 with ln(CRP) = 3.07. In the SPT, however, baseline CRP levels are higher; an average SPT child has a baseline ln(CRP) = 3.74, peaking at approximately day 2 with ln(CRP) = 4.17 but quickly decreasing with time. In the SSM, baseline CRP levels were similar to those in the SPT (ln(CRP) = 3.36 for an average child with septic shock or MODS); contrary to the SPT, however, the levels reached the maximum slightly later, approximately day 4 (ln(CRP) = 4.43), and decreased less rapidly.

We believe that the differences in baseline values of CRP levels of the NIN patients versus the other two groups are the result of the study design. We defined day 0 in a slightly different way for the former group; thus, the onset of the CRP level increase can result from different factors in the infectious groups (SPT and SSM patients) and in the noninfectious group. As Figure 2 illustrates, absolute values of CRP are higher in the SPT and SSM, peaking at days 2 to 4, compared with the values in the NIN. These findings are consistent with those of other studies [13,14]. We, however, present another consideration: the rate of decreasing CRP levels is slower in the shock group than in the septic group.

### Full model B

The second final model (full model B) includes an additional predictor: a diagnosis dichotomy (internal or surgical). Including this additional predictor, we obtained another model with a slightly lower score of information criteria (Akaike's information criteria, 1,539.6 (model A) compared with 1,544.0 (model B); and Schwarz's information criteria, 1,578.7 (model A) compared with 1,580.5 (model B). This full model B indicates that CRP levels are higher in surgical (or traumatic) patients than in patients with an internal diagnosis. Owing to an insignificant interaction of diagnosis with the septic category as well as with time, we can conclude that the effect of surgical diagnosis is, on a logarithmic scale, approximately the same for each septic category and over time. The magnitude of this additional elevation in surgical patients therefore does not depend on septic condition. Computing the model equations (Table 6), we can see that the differences in ln(CRP) levels at their peaks between an average child with a nonsurgical diagnosis and one with a surgical diagnosis are 2.54 versus 3.2, 4.01 versus 4.67, and 4.3 versus 4.96 for the NIN, SPT, and SSM, respectively.

### C-reactive protein and other proinflammatory markers

Many authors target finding proinflammatory markers of infection and SIRS other than the CRP, such as PCT, IL-1, IL-6, or TNFα [5-9,13,15,16]. Some of these studies [13,15,16] compared PCT levels with CRP levels in septic patients, suggesting that PCT can be a more reliable marker than CRP. Unfortunately, none of these studies used multilevel modeling for the statistical analysis, which could have been beneficial in the evaluation of dynamic changes in proinflammatory markers.

In our study, we demonstrated that, over time, septic condition and trauma influence CRP blood levels in children. Hence, comparison of CRP and other proinflammatory markers such as PCT can be difficult because of their different kinetics and because of the heterogeneity among participants (for example, different medical and surgical conditions) in different studies. Even obtaining blood levels of both markers at the same time point would therefore, in the clinical sense, result in different values. In our study, we found only a weak correlation (*R *= 0.34, *P *= not significant) between CRP and PCT blood levels, supporting these ideas. This comparison was performed on a limited group of 20 patients with available data for both CRP and PCT blood levels at the same time points. Similar findings, in the context of time and different stimuli resulting in PCT elevation, may be apparent from other studies [25,26]. In designing similar studies, therefore, the dynamics of different markers as well as various factors stimulating immune response should be accounted for to improve the diagnostic and prognostic values of these markers.

### Sources of variability

The presented findings raise questions about causes. We believe that, in patients with septic shock or MODS, the stimulus inducing CRP production lasts longer. The decrease in CRP levels is therefore slower in these patients. Other factors in addition to shock and organ dysfunction, however, may cause the prolonged elevation of CRP (for example, higher risk of secondary infection or difficult elimination of present infection in these severe conditions), and these still remain to be explored. The SSM included four patients with cancer, a factor that may also play a role [16]. On the other hand, in septic patients – in whom we assume that infection is the main factor inducing CRP production – CRP levels can quickly drop after successful treatment. These considerations are consistent with the physiology of the immune response [27,28]. The additional increase of CRP in surgical patients indicates that another factor influences CRP production. With respect to our findings, traumatic insult or surgical intervention may cause increased CRP production; within the 10-day time period in this study, the increased production was constant over time (on logarithmically transformed data).

Comparing the variances in the two final models with the unconditional growth model, we can see that including either the septic category as a predictor (model A) or the septic category with diagnosis as predictors (model B) both reduces the variability of baseline CRP values and their rates of change by about 20% and 1.5%, respectively. Because the variation remains significant in both models, other predictors still remain to be found.

### Usefulness of the modeling approach

Various diagnoses of patients included in our sample as well as other factors introducing heterogeneity into the sample (for example, age, localization of infection, and so on) preclude the model itself from a direct clinical use. This was not, however, the main goal. We particularly wanted to show a new method for analyzing longitudinal data such as these, and how to interpret the results. Other methods and/or models might be used but we consider the presented models both easy and sufficiently informative.

### Limitations

As mentioned above, other predictors could possibly explain another part of the remaining variation or the overall variance more comprehensively. These other predictors could be age, sex, more specifically categorized diagnosis, localization of infection, more accurately defined organ dysfunction (Sequential Organ Failure Assessment score), and possibly other factors. We could not perform a more precise analysis based on the abovementioned factors because of the relatively small patient groups; patient numbers in each category would have been quite small, making a correct, unbiased analysis impossible.

As Figure 1 shows, CRP levels (in some patients) remain elevated or are even increased in the septic group. This phenomenon could have been caused by secondary infection, insufficiency of diagnostic criteria or eligibility criteria for the study, or other unknown reasons. Moreover, many patients had incomplete data records, as shown by short lines in Figure 1. These factors could lead to a decreased accuracy of the models used. On the other hand, by knowing these negatively acting factors as well as other important predictors, we may arrive at more accurate models with more precise predictive capability.

From the statistical point of view, a different model to that presented (linear with a quadratic term) may be more suitable; for example, a nonlinear model. But we think the simpler linear model we presented here is easier to interpret and, considering the research questions, is sufficient for analyzing the data. Another problem, however, arises from the data. We can see that the numbers in some patient groups are quite small. We had to deal with the data we had available. There were no more patients in the most severe category (fortunately for the patients). The estimates, however, can be biased by this fact. To be somewhat sure of the results, we performed the following procedure. We performed the analysis without both effects (SEP, DG) together; that is, we performed the analysis separately with SEP (which is actually model A) and DG, and based on these analyses we could draw the same conclusions concerning SEP and DG as we already had done.

Since the analysis was intended as exploratory, we consider the results sufficiently clear. To explore the variance heterogeneity we performed the M Box test, which tests the homogeneity of a covariance matrix [29]. This test was performed on a restricted group of patients with sufficient data in the first six time points (41 subjects in four groups) and we obtained *P *= 0.132. We could not perform the test in all groups due to the lack of the data but we think that the model analyses presented here could be performed assuming that the covariance matrix did not significantly differ among the analyzed groups.

Since the analysis was performed on the whole sample data collected, the model needs a validation set for model validation. The present paper, however, was intended only as an exploratory analysis that should give the first insight into the data.

Because the study was retrospective, and due to the limitations mentioned above, we intend to perform a prospective study to verify these findings in a larger cohort of patients. Nevertheless, this study poses novel considerations based on simple monitoring of dynamic changes of blood CRP levels in children with sepsis, with results that prove worthy of further investigation.

## Conclusion

Our results suggest that the more severe the systemic reaction to the insult, the higher and the more prolonged the CRP levels. Moreover, in patients with the most severe conditions, such as septic shock and MODS, the rate of decrease of CRP levels was less rapid than in common septic patients. We demonstrated that septic patients after trauma or surgical intervention have higher CRP levels compared with patients with other diagnoses. Following the overall dynamics of CRP, blood levels can improve the prognostic and diagnostic value of CRP as a marker of sepsis severity compared with consideration of its values separately at single time points. In conclusion, multilevel modeling is a novel technique for analyzing longitudinal data that can be applied successfully in CRP level monitoring.

## Key messages

• Mixed models and multilevel modeling are suitable for analyzing CRP longitudinal data.

• The dynamics of CRP blood levels in children is influenced by the septic condition and trauma.

• CRP levels decrease less rapidly in children with more severe septic conditions (septic shock, MODS) in contrast to those with sepsis/severe sepsis. CRP levels reach lower values in patients with SIRS than in those with sepsis, septic shock, or MODS.

• CRP levels are higher in children after immediately preceding trauma/surgery intervention than in those without this condition, and the decrease is comparable in both surgical and nonsurgical groups of patients.

• We provided a novel sight of inflammatory markers and pointed out the need for considering these findings in designing studies comparing the usefulness of the markers.

## Abbreviations

CRP = C-reactive protein; DG = diagnosis effect; IL = interleukin; MODS = multiple organ dysfunction syndrome; NIN = noninfectious group; PCT = procalcitonin; SEP = effect of septic category; SIRS = systemic inflammatory response syndrome; SPT = septic group; SSM = shock and multiple organ dysfunction syndrome group; TNF = tumor necrosis factor.

## Competing interests

The authors declare that they have no competing interests.

## Authors' contributions

MK collected data, performed the statistical analyses, participated in the design of the study, and composed the manuscript. MF interpreted the clinical characteristics of patients. LE helped with designing the study. NK helped with patient classification and proofread the manuscript. JM designed and supervised the study and wrote the manuscript. All authors read and approved the final manuscript.
